# Lipid dynamics at dendritic spines

**DOI:** 10.3389/fnana.2014.00076

**Published:** 2014-08-08

**Authors:** Carlos Gerardo Dotti, Jose Antonio Esteban, María Dolores Ledesma

**Affiliations:** Centro Biología Molecular Severo Ochoa, CSIC-UAMMadrid, Spain

**Keywords:** dendritic spines, cholesterol, sphingolipids, phosphoinositides, glutamate receptors, synaptic plasticity

## Abstract

Dynamic changes in the structure and composition of the membrane protrusions forming dendritic spines underlie memory and learning processes. In recent years a great effort has been made to characterize in detail the protein machinery that controls spine plasticity. However, we know much less about the involvement of lipids, despite being major membrane components and structure determinants. Moreover, protein complexes that regulate spine plasticity depend on specific interactions with membrane lipids for proper function and accurate intracellular signaling. In this review we gather information available on the lipid composition at dendritic spine membranes and on its dynamics. We pay particular attention to the influence that spine lipid dynamism has on glutamate receptors, which are key regulators of synaptic plasticity.

## Introduction

At most excitatory synapses in the Central Nervous System, presynaptic boutons synapse onto small membrane protrusions that emerge from the dendritic shaft: the dendritic spines. Changes in dendritic spine number, size and shape contribute to determine the strength of excitatory synaptic transmission (Yuste and Bonhoeffer, 2001; Carlisle and Kennedy, 2005). The remodeling of these membrane protrusions in response to stimuli depends on lipids, which are major components of the membrane with the ability to shape it and modify protein activities within. However, only recently the contribution of spine lipids has attracted similar attention to that of spine proteins. Pioneer work showing the requirement of glial cholesterol for synapse formation (Mauch et al., 2001) and the elimination of spines upon reduction of cholesterol or sphingolipids (Hering et al., 2003) triggered research in this field. Technical progress facilitates today the not so long ago impossible analysis of the subtle changes in lipid composition and of the topographical distribution of individual lipid species in cellular compartments. Probes have been developed to label lipid molecules such as new generation fluorescent tags (Eggeling et al., 2009) or modified toxins with specific lipid binding abilities such as the theta-toxin or lysenin, which bind cholesterol or sphingomyelin, respectively (Abe et al., 2012). These probes together with advanced microscopy techniques that achieve sub-diffraction optical resolution (i.e., near-field scanning optical microscopy (NSOM), photoactivated localization microscopy (PALM) stochastic optical reconstruction microscopy (STORM) or stimulated depletion (STED) fluorescent microscopy) allow the direct observation of the nanoscale dynamics of membrane lipids in a living cell (Eggeling et al., 2009; van Zanten et al., 2010; Castro et al., 2013). As we gain insight on how lipids and their metabolic enzymes regulate dendritic spine shape and protein function their importance is confirmed and strengthened. We aim here to review this knowledge focusing the attention on the dynamic lipidomics of dendritic spines. We will also discuss about how this influences synaptic plasticity through the modulation of glutamate receptors of the AMPA and NMDA-type (AMPARc and NMDARc). These receptors are instrumental to elicit Long Term Potentiation (LTP) and Long Term Depression (LTD), which are considered the molecular mechanisms underlying learning and memory (Neves et al., 2008; Collingridge et al., 2010).

## Lipid composition at dendritic spines

A relevant question about spine physiology is whether spine membrane lipid composition and organization is different to that of the dendritic shaft membranes from which these protrusions emerge. A systematic analysis of spine lipid composition is lacking due to technical limitations. However, accumulating evidence indicates it differs from that of the shaft. This raises questions such as why this specificity is necessary and how it is achieved, maintained or modulated upon stimuli. Until now, most of the information on synaptic lipid composition comes from the biochemical analysis of synaptosomal preparations. Functional studies have also highlighted the relevant contribution of certain lipids to spine physiology. From these two types of approaches we now know that cholesterol and sphingolipids are enriched in spines. Because of their chemical affinity these lipids form highly dynamic and heterogeneous membrane nanodomains, the so called rafts, which can be stabilized to form larger platforms by protein-protein or protein-lipid interactions (Pike, 2006). Rafts compartmentalize cellular processes contributing to the accurate spatial and temporal organization of molecules required at dendritic spines (Allen et al., 2007). Neurotrophin and neurotransmitter receptors (NTRcs) are recruited from extrasynaptic to synaptic sites through association to lipid rafts, which reduce receptor lateral mobility at the synaptic space (Nagappan and Lu, 2005; Fernandes et al., 2010). In fact, the post synapse has been proposed as a lipid raft-enriched territory and certain key structural proteins such as the postsynaptic density protein 95 (PSD95) as well as AMPARc dynamically associate to these domains (Perez and Bredt, 1998; Suzuki, 2002; Hering et al., 2003; Suzuki et al., 2011). The tight control of the turnover of phosphoinositides and their derivatives plays also a central role in spine plasticity. We next describe data available on the presence of the aforementioned lipids in spines and on their contribution to spine physiology. Hopefully, the already mentioned imaging techniques based on advanced lipid probes and super-resolution microscopy together with most sensitive quantitative measurements (i.e., liquid chromatography coupled with tandem mass spectrometry) would contribute to more precisely define the lipid composition of spines and its changes in real time in living cells.

### Cholesterol

Pharmacological extraction of cholesterol or inhibition of its synthesis led to the disappearance of dendritic spines in cultured hippocampal neurons, probably mediated by disruption of the actin cytoskeleton (Hering et al., 2003). This finding defined cholesterol as a core component of spines. A series of functional reports have demonstrated the relevance of this lipid for synaptic plasticity. Pioneer work showed that acute cyclodextrin-mediated removal of membrane cholesterol blocks LTP in the hippocampus (Koudinov and Koudinova, 2001; Frank et al., 2008). On the other hand, excitatory neurotransmission, chronic and acute, induces cholesterol loss from synapses, which is recovered after stimuli (Sodero et al., 2011, 2012). In the aging brain, lifelong lasting synaptic activity and concomitant metabolic stress contributes to a moderate but irreversible loss of membrane cholesterol (Sodero et al., 2011), which is thought to underlie cognitive deficits present at this stage of life (Martin et al., 2014). In agreement, cholesterol replenishment restores LTD in hippocampal slices from aged mice and improves their learning and memory abilities (Martin et al., 2014). The molecular mechanisms by which cholesterol influences postsynaptic plasticity are just beginning to be understood. Thus, impaired LTD in the old produced as consequence of lower membrane cholesterol can be explained, to a certain extent, by sustained activity of the PI3K/Akt pathway, in turn leading to inactivation of GSK3β and reduced AMPAR internalization (Martin et al., 2014). The broad effect of cholesterol on the biophysical properties of the membrane bilayer (i.e., viscosity, Renner et al., 2009) may affect the molecular flow in and out of synapses and therefore the mobility and interactions of NTRc (Fantini and Barrantes, 2009). Changes in the amount of cholesterol at spines might also exert functional effects through cholesterol metabolites. Thus, the most abundant in the brain, 24(S)-hydroxycholesterol, is a potent and selective positive modulator of NMDARc and enhances LTP (Paul et al., 2013).

### Sphingolipids

Long term stability and dynamic changes in dendritic spines are intimately linked to the actin cytoskeleton, which is particularly enriched in these structures (Hotulainen and Hoogenraad, 2010; Koleske, 2013). Changes in the amount of filamentous actin (F-actin) mediate long-lasting alterations in spine size and synaptic efficacy. The repetitive firing of synapses that occurs during high-frequency stimulation to induce LTP, promotes actin polymerization and spine enlargement (Matsuzaki et al., 2004; Okamoto et al., 2004). Conversely, treatment that weakens synaptic efficacy, such as low-frequency stimulation that results in LTD, causes F-actin loss and dendritic spine shrinkage (Okamoto et al., 2004; Zhou et al., 2004). Enlargement and shrinkage of the spine requires the coordination of the actin cytoskeleton with the membrane. Recent work shows that the most abundant sphingolipid in neuronal membranes, sphingomyelin (SM), plays a relevant role in the spine membrane-cytoskeleton crosstalk by modulating membrane binding and activity of main regulators of the actin cytoskeleton at synapses: the Rho GTPases. Hence, high SM levels lower the amount of type I metabotropic glutamate receptors (mGluRs) at the cell surface impairing membrane attachment, and therefore activity, of the small GTPase RhoA concomitantly with the inhibition of its effectors ROCK and Profilin IIa. This seems to be the mechanism leading to reduced F-actin content and smaller size of spines in mice lacking the acid sphingomyelinase (ASM) gene (Arroyo et al., 2014). Conversely, low SM levels in postsynaptic membranes are responsible for the enhanced activity of the RhoA-ROCK-Profilin I pathway resulting in increased actin polymerization and dendritic spine size in mice lacking the actin related protein WIP (Franco-Villanueva et al., 2014). Moreover, the dynamic partitioning of RhoA into raft membrane domains, which is enhanced upon stimuli, is dependent on the maintenance of appropriate SM levels at the synaptic membrane (Franco-Villanueva et al., 2014).

Ceramide is another major sphingolipid contributing to spine plasticity by virtue of its capacity to favor membrane fusogenicity promoting receptor clustering (Krönke, 1999). The rapid generation of ceramide modulates excitatory postsynaptic currents by controlling the insertion and clustering of NMDARc (Wheeler et al., 2009). In agreement, direct additions of synthetic cell-permeable ceramide analogs increase excitatory postsynaptic currents without affecting presynaptic plasticity (Coogan et al., 1999; Fasano et al., 2003). The ceramide-associated enhancement of excitatory currents is often transient and is followed by sustained depression of excitatory postsynaptic currents (Coogan et al., 1999; Yang, 2000; Davis et al., 2006). These findings support complex roles for the spatial and temporal production of ceramide in regulating neuronal excitability. In addition, ceramide contributes to spine maturation by promoting the transformation of dendritic fillopodia into mature spines (Carrasco et al., 2012).

### Phosphoinositides and derivatives

Despite phosphoinositides (PIPs) are minor components of synaptic membranes, their exceptional high rate of metabolic turnover and their compartmentalization make them key players in postsynaptic excitability (Hammond and Schiavo, 2007). The presence at dendritic spines of the enzymes that interconvert different PIPs supports a relevant role for these lipids in the dynamics of these structures.

Continuous synthesis and availability of phosphatidylinositol(3,4,5) triphosphate (PIP3) at the postsynaptic terminal is necessary for sustaining synaptic function in rat hippocampal neurons (Arendt et al., 2010). PIP3 shows greater accumulation in spines than in dendritic shafts under basal conditions. Interestingly, glutamate stimulation promotes spine enlargement and the appearance of filopodia-like protrusions (spinules) projecting from spines (Richards et al., 2005) to which PIP3 redistributes (Ueda and Hayashi, 2013). Consistent with a key role for PIP3 in spinule formation, blockage or inhibition of this lipid prevent their appearance (Ueda and Hayashi, 2013). The biological significance of spinules is not yet established. However, trans-endocytosis of these protrusions by presynaptic buttons may aid postsynaptic membrane remodeling by removing the excess membrane at postsynaptic sites (Spacek and Harris, 2004). It was also proposed that PIP3 signaling at spinules enables new synapses to form with functional presynaptic boutons contributing to the change in synaptic connectivity. PIP3 could also mediate membrane-cytoskeleton crosstalk at spines by virtue of its capacity to regulate the activity of multiple Rho GTPase effectors (Yin and Janmey, 2003). Additionally, PIP3 is the upstream regulator of the Akt-mTOR pathway, which signals activity-dependent regulation of protein synthesis (Kelleher et al., 2004) and participates in dendritic and spine morphogenesis (Kumar et al., 2005).

**Figure 1:**
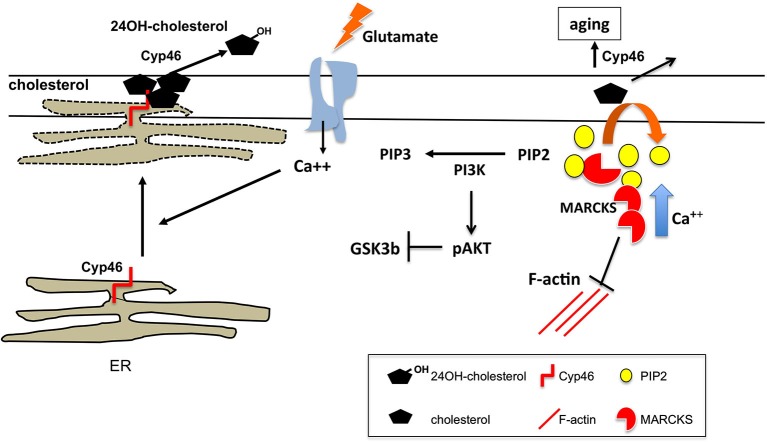
**Cholesterol regulation at spines upon glutamate stimulation**. Acute glutamate stimulatory conditions lead to loss of membrane cholesterol. The mechanism proposed involves glutamate induced rise in intracellular Ca++ leading to the approximation/apposition of ER membranes to the synaptic plasma membrane. This allows Cyp46A1, whose active site is in the lumenal side of the ER, to oxidize cholesterol present in the exoplasmic leaflet that is released as hydroxycholesterol. In the aging context, constitutive high intracellular calcium and irreversible cholesterol loss due to lifelong lasting synaptic activity leads to reduced membrane-associated MARCKS, which affects synaptic plasticity by several mechanisms: (1) impaired MARCKS-mediated actin dynamics; (2) reduced membrane clustering of PIP2; and (3) high PI3K activity resulting in reduced glutamate-mediated Akt dephosphorylation and GSK3β activation. The later contributes to the impaired AMPARc internalization and LTD in aged neurons.

Appropriate levels and clustering of phosphatidylinositol(4,5) diphosphate (PIP2) at the postsynaptic membrane, which are modulated by the activities of Phospholipase *γ* (PLC*γ*) and PIP5K, are important for synaptic plasticity, both LTP (Trovò et al., 2013) and LTD (Unoki et al., 2012). The PIP2-clustering molecule myristoylated alanine-rich C kinase substrate (MARCKS) critically contributes to this requirement. The effector domain of MARCKS reversibly sequesters PIP2 on the plasma membrane, which can be released in response to local increases in intracellular calcium (McLaughlin and Murray, 2005). Low levels of this protein, leading to PIP2 paucity at the membrane, promote the age-related impairment of synaptic plasticity (Figure 1). Hence, its overexpression in the hippocampus of old mice or intraventricular perfusion of MARCKS peptide result in enhanced LTP and improved memory (Trovò et al., 2013). On the other hand, MARCKs appears to be a key molecule in spine morphogenesis promoting the transition from thin immature dendritic spines to larger, more stable mushroom by controlling actin cytoskeleton (Calabrese and Halpain, 2005). In agreement, MARCKs deficient mice show impaired LTP and spatial cognition (McNamara et al., 1998; Hussain et al., 2006). Association of MARCKS to the membrane is necessary for its ability to crosslink F-actin (Calabrese and Halpain, 2005). Membrane levels of cholesterol, to which MARCKs can bind, would mediate this association. Evidence suggests that indeed defective MARCKs-induced PIP2 clustering in old synaptic membranes responds to the reduction of cholesterol levels during aging (Trovò et al., 2013; Martin et al., 2014; Figure 1).

Besides PIPs themselves, PIP derived second messengers such as Diacylglycerol (DAG) and 1,4,5-triphosphate (IP3) generated by the hydrolysis of PIP2 by PLC, are also important in dendritic spine organization and function. Unlike IP3, which is released into the spine cytoplasm, DAG is embedded in the membrane and recruits and activates DAG effectors (among them PKC), which have been involved in spine maintenance (Brose et al., 2004). DAG molecules are produced in dendritic spines through activation of postsynaptic receptors, including those of the NMDA type. It has been proposed that the rapid and focal generation of DAG, together with ceramide, triggers the fusion of vesicles and insertion of NMDARc subunits into lipid rafts. Based on the biophysical properties of DAG, it is likely that the generation of this lipid serves to destabilize the membranes to create fusion points at the postsynapse (Wheeler et al., 2009). In turn, phosphatidic acid (PA) that is generated by DAG phosphorylation, also regulates spines together with its effectors. Among them, the alpha-p-21-activated kinase PAK1 promotes spine formation by stabilizing actin filaments through myosin phosphorylation (Zhang et al., 2005).

## Dynamism of the lipid composition at dendritic spines: the role of lipid metabolic enzymes

The aforementioned evidences highlight not only the wealth and specificity of the lipid composition at spines but also its dynamism and panoply of biological roles. The question remains on how this lipid composition is achieved and maintained, or changed, in different physiological situations. While still being an open question, local recruitment and removal of the different metabolic enzymes is a reasonable possibility. In fact, a number of these enzymes have been localized in dendritic spines, change their activity upon synaptic stimuli and/or have the ability to modulate NTRc trafficking and function. While the recruitment of certain enzymes occurs in response to stimuli others are constitutively present at postsynapses. In the following section we describe examples of the contribution of lipid metabolic enzymes to spine physiology.

### Cholesterol 24-hydroxylase

The cytochrome P450 enzyme, cholesterol 24-hydroxylase (Cyp46A1) is selectively expressed in the brain, where it is responsible for cholesterol oxidation and eventual excretion to the general circulation (Lund et al., 1999). Pyramidal neurons of the hippocampus and cortex, Purkinje cells of the cerebellum, and hippocampal and cerebellar interneurons show particularly high levels of this enzyme, which is preferentially localized in the endoplasmic reticulum (ER) of dendrites and cell bodies (Ramirez et al., 2008). Cyp46A1 contribution to synaptic plasticity is supported by the observations that mice lacking this enzyme present impaired learning and hippocampal LTP (Kotti et al., 2006) while mice overexpressing human Cyp46A1 present improved spatial memory (Maioli et al., 2013). Increased levels of Cyp46A1 parallel the mild but significant cholesterol reduction in membranes of the hippocampus of old rodents and of hippocampal neurons aged in culture (Martin et al., 2008; Sodero et al., 2011). In turn, knockdown of the enzyme prevents glutamate-mediated cholesterol loss (Sodero et al., 2012), supporting a cause-effect relationship between cholesterol reduction in the aged and Cyp46A1 increased activity. Biotinylation, electron microscopy and TIRF analysis indicated that Cyp46A1 is present in spines of hippocampal neurons and that, upon stimulation, a close approximation occurs between the site of residence of the enzyme (the ER) and the plasma membrane, suggesting that this could be the mechanism by which cholesterol is removed from the plasma membrane (Sodero et al., 2012). The increase in surface Cyp46A1 after stimulation supports this notion, and the higher production of its metabolite 24S-hydroxycholesterol in stimulated neurons indicates that the pool of the enzyme that increase at the plasma membrane is active. Not surprisingly for an ER-resident protein, the process of Cyp46A1 mediated cholesterol loss requires high levels of intracellular Ca^2+^ and a functional ER-plasma membrane communication via the stromal interaction molecule 2 (STIM2; Sodero et al., 2012). It has been proposed that high levels of Ca^2+^ elicit such communication bypassing the Golgi apparatus. This process would be consistent with the observation that the distance between the ER and the plasma membrane can be as small as 10 nm (Pichler et al., 2001; Lebiedzinska et al., 2009) and that NMDARc stimulation produces a transient and reversible fission of ER tubules (Kucharz et al., 2009). This mechanism provides an efficient, temporally and spatially controlled, mean to change postsynaptic membrane lipid composition (Figure 1). Whether lipid metabolic enzymes other than Cyp46A1 follow the same mechanism is unknown.

### Neutral sphingomyelinase-2

**Figure 2:**
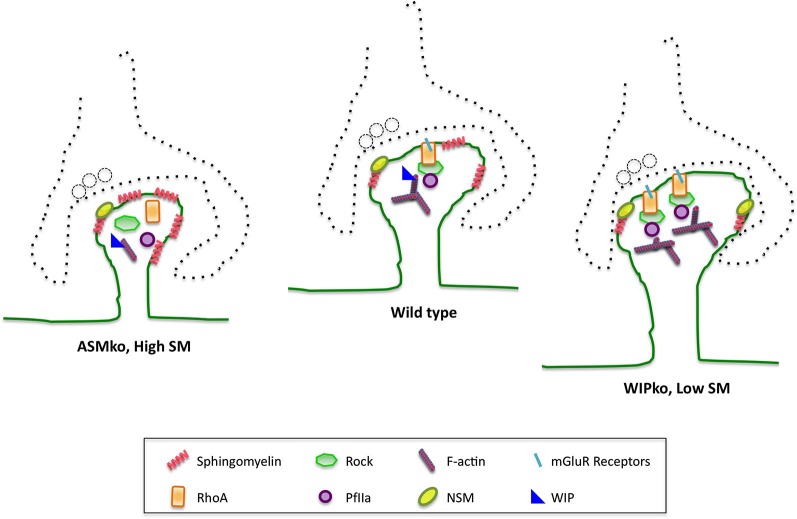
**SM and its catabolic enzymes in dendritic spine physiology and pathology**. SM levels at the postsynaptic membrane modulate membrane binding and activation of the small GTPase RhoA, which in turn modulates F-actin content through its effectors ROCK and profillin 2A. This molecular mechanism controls dendritic spine size. The SM catabolic enzymes ASM and NSM are involved in this process. Hence, mice lacking ASM show abnormally high SM levels in their postsynaptic membranes that lower the amount of cell surface mGluRs. This impairs RhoA membrane binding and pathway activation, which diminish spine F-actin content and size. These anomalies can be corrected by NSM activation. Conversely, mice lacking the actin related protein WIP show constitutively active NSM leading to reduced SM levels, which enhance RhoA membrane binding and pathway activation resulting in bigger dendritic spines with higher F-actin content. Spine anomalies could explain the cognitive deficits observed in ASMko mice, which are a model for Niemann Pick disease type A, and those of individuals carrying mutations in the region encoding for WIP.

Among the sphingolipid metabolic enzymes the Neutral sphingomeylinase-2 (NSM) has been directly related to spine size and to postsynaptic function. NSM is the main responsible of SM degradation and conversion to ceramide at the plasma membrane (Stoffel, 1999). Its rapid kinetics and location enriched in hippocampus made it a likely candidate to modulate plasticity (Hofmann et al., 2000). In support, abundant NSM has been found in synaptic membranes (Arroyo et al., 2014). The rapid generation of ceramide by NSM modulates excitatory postsynaptic currents by controlling the insertion and clustering of NMDARc (Wheeler et al., 2009). In addition recent work shows the ability of this enzyme to modulate spine actin cytoskeleton. Hence, activation of NSM corrects the abnormally low size and F-actin content of dendritic spines in mice lacking the acid sphingomyelinase, which present high SM synaptic levels, by enhancing the RhoA pathway (Arroyo et al., 2014). Conversely, NSM inhibition restores normal RhoA activity and diminishes the abnormally increased size and F-actin levels of spines in neurons of mice lacking WIP (Franco-Villanueva et al., 2014; Figure 2). This actin-related protein has the ability to sense the levels of F-actin to which it can bind. It has been proposed that WIP modulates SM amount at the spine membrane by RhoA mediated transcriptional control of the NSM, thus controlling the spine response to actin polymerization stimuli (Franco-Villanueva et al., 2014). This places NSM at a key position to mediate membrane-cytoskeleton crosstalk at spines. It remains to be determined whether the enzyme levels and activity could be modulated at synapses by a local transcriptional control mechanism. The observation that activation of NSM with dexamethasone corrects actin related anomalies in isolated synaptosomes argues in favor of this possibility (Arroyo et al., 2014). If true, the question arises on whether, similar to those of many actin related proteins, mRNAs of lipid metabolic enzymes are present at synapses to facilitate the immediate spine remodeling in response to stimuli. Alternatively, NSM would translocate to the synaptic plasma membrane upon stimulation. This is supported by evidences in non-neuronal cells showing that a stress-mediated PKC mechanism induces the appearance of the enzyme at the plasma membrane (Clarke et al., 2008). The possibility that not only NSM but also its analog enzyme, ASM, regulates SM levels at the postsynaptic membrane is not yet clarified. The presence of ASM in spines has not been reported. However, the observations that lack of this enzyme leads to reduced spine size and F-actin content (Arroyo et al., 2014) and that it can function at neutral pH (Schissel et al., 1998) or in acidified microenvironments that may exist at the cell surface (Bourguignon et al., 2004), support the regulated action of the two sphingomyelinases to control spine SM levels and actin cytoskeleton (Figure 2).

### Carnitine palmitoyltransferase 1C (CPT1C)

The brain specific isoform of Carnitine Palmitoyltransferase 1 (CPT1C), modulates ceramide levels in hippocampal neurons where it is especially enriched (Carrasco et al., 2012). CPT1C is located in the ER and has been recently found inside dendritic spines. The mechanism by which this enzyme, which facilitates fatty acid transport across intracellular membranes (Wolfgang et al., 2006), modulates ceramide levels is still unknown. Activation of *de novo* synthesis of the lipid has been discarded. Instead, CPT1C may influence the generation of ceramide from the sphingosine pool through the salvage pathway and/or on its degradation. CPT1C deficiency increases immature filopodia and reduces mature mushroom and stubby spines, while not affecting total spine number or spine head area. These effects on spine maturation can be restored by ceramide addition (Carrasco et al., 2012). Consistent with the role of CPT1C on the transformation of dendritic filopodia into mature spines, mice lacking this enzyme show defects in hippocampus dependent learning abilities (Carrasco et al., 2012).

### Phosphoinositide metabolic enzymes

**Figure 3:**
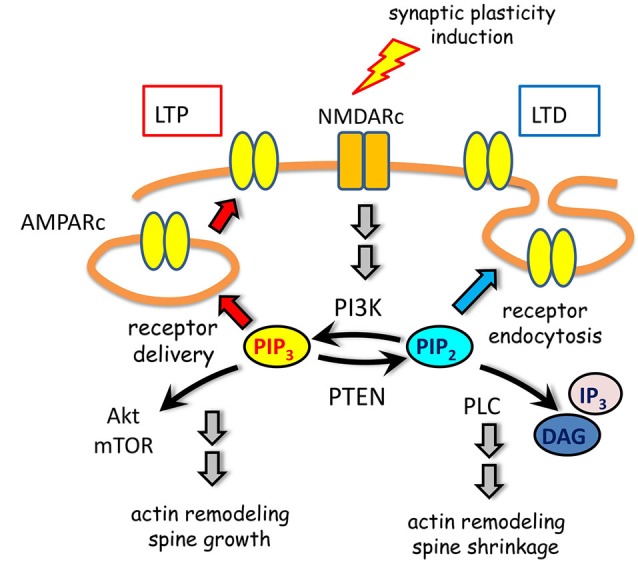
**PIP metabolism in spine plasticity**. Activation of NMDARc modulate AMPARc trafficking through spatially and timely controlled activity of PIPs and their metabolic enzymes. On one hand, PI3K association with AMPARc is required for receptor cell surface delivery during LTP. On the other hand, PTEN activity leading to PIP3 downregulation promotes migration of AMPARc from the postsynaptic density to the perisynaptic membrane. This depresses AMPARc synaptic responses by promoting receptor endocytosis during LTD. Moreover, signaling pathways initiated by PIP3 or PIP2, in which Akt/mTOR, DAG and IP3 are involved, contribute to actin remodeling and spine changes in size.

Several enzymes tightly control PIP turnover at dendritic spines (Figure 3). Biochemical and imaging experiments demonstrated that the phosphatase and tensin homolog deleted on chromosome ten (PTEN), which converts PIP3 into PIP2 (Maehama and Dixon, 1999), is recruited to dendritic spines upon NMDARc but not AMPARc activation (Jurado et al., 2010). NMDARc activation triggers a biphasic regulation of PTEN mobility in dendritic spines. First, there is a rapid and transient increase in mobility independent from PTEN interactions through its PDZ motif. A longer-lasting and PDZ-dependent anchoring of PTEN to the postsynaptic density follows this phase. This regulated mechanism of recruitment of PTEN may provide means to achieve synapse-specific modulation of PIP3 signaling during plasticity. The enhancement of PTEN lipid phosphatase activity is able to drive depression of AMPARc-mediated synaptic responses (Jurado et al., 2010). Consistently, mice with altered PTEN expression show multiple impairments in synaptic function including LTP and LTD (Wang et al., 2006; Fraser et al., 2008).

The class I phosphatydilinsositol-3-kinase (PI3K) constitutively localizes at synapses by means of a direct interaction between its p85 subunit and the AMPARc (Man et al., 2003). By converting PIP2 into PIP3 this kinase ensures the delivery of new AMPARc into spines in response to NMDARc activation (Man et al., 2003) and the maintenance of AMPARc clustering at the postsynaptic membrane (Arendt et al., 2010; Figure 3).

The major PIP2 producing enzyme in the brain, the phosphatidylinositol-4-phosphate 5-kinase*γ*661 (PIP5K*γ*661), becomes dephosphorylated and associates with the clathrin adaptor protein complex AP-2 at postsynapses upon NMDA receptor activation (Unoki et al., 2012). This event is necessary to elicit AMPARc endocytosis and LTD. PLC, which catalyzes hydrolysis of PIP2, is enriched in spine heads and primarily localized in a thin border around the postsynaptic density (Yoshida et al., 2006). PLC activity is enhanced by NMDARc stimulation and its blockage impairs LTD (Reyes-Harde and Stanton, 1998). This activity is required for changes in postsynaptic structure by depolymerizing spine actin and decreasing PSD95 levels. This in turn promotes AMPARc internalization during LTD (Horne and Dell’Acqua, 2007). PLC has been also related to the termination of synaptic stimuli. It has been proposed that lipid signals (i.e., 2-arachidonoylglycerol) generated at dendritic spines by PLC activity, upon activation of type I metabotropic glutamate receptors (mGluRs), diffuse across the synaptic cleft and activate cannabinoid receptors, reducing presynpatic Ca^2+^ channel activity and inhibiting further glutamate release (Chevaleyre et al., 2006).

### DAG kinases

Evidence accumulates in support of the notion that DAG-metabolizing enzymes DAG kinases (DGK), which are coupled to synaptic scaffolding proteins, tightly control synaptic DAG concentrations (Kim et al., 2010). Activation of NTRc, including mGluR and NMDARc, induce the production of DAG and its phosphorylation by DGKs, converting DAG into PA. The DGKζ isoform has been critically involved in spine maintenance. DGKζ is targeted to excitatory synapses through its direct interaction with the postsynaptic scaffold protein PSD-95. Overexpression of DGKζ in cultured neurons increases the number of dendritic spines in a manner requiring its catalytic activity and PSD-95 binding. Conversely, DGKζ knockdown reduces spine density (Kim et al., 2009). In agreement, mice deficient in DGKζ expression show reduced spine density and excitatory synaptic transmission. Time-lapse imaging indicates that DGKζ is required for spine maintenance but not formation (Kim et al., 2009). It has been proposed that DAG and PA signaling pathways are integrated within synaptic multi-protein complexes that intersect with small GTPases controlling actin cytoskeleton (Tada and Sheng, 2006; Kim et al., 2010).

## Influence of membrane lipids on glutamate receptor function at dendritic spines

Modifications in the number and complement of glutamate-sensing receptors in the postsynaptic membrane are key mechanisms to adjust strength in excitatory synapses. AMPA and NMDA-type glutamate receptors are ligand-gated ion channels critical for synaptic plasticity (Barria and Malinow, 2002; Malinow and Malenka, 2002; Washbourne et al., 2004). Although the identification and characterization of proteins involved in the regulation of these receptors has been a productive area of research, there has been less progress in understanding how changes in membrane lipids affect their function. Lipids could modulate glutamate receptor affinity or capacity to bind their ligands by influencing receptor conformation, orientation, subunit composition or oligomerization. Lipids could as well alter the properties of the channels themselves. Lateral movement and endo-exocytic trafficking of NTRcs are essential for glutamate signaling. Lipids are also good candidates to regulate these membrane dependent events. Although we still lack accurate information about the differences in lipid composition between synaptic and extrasynaptic sites, specific lipid changes (i.e., cholesterol or PIP3 loss) affect the synaptic but not the extrasynaptic pool of AMPARc (Arendt et al., 2010; Martin et al., 2014). Hence, lipids could contribute to the balance between synaptic and extrasynaptic glutamate receptor activity, which is key for synaptic plasticity. Accumulating evidence supports the view that lipid rafts provide both a spatial and a temporal meeting point for receptors and proteins involved in a common pathway facilitating their intracellular signaling (Allen et al., 2007). Supporting the relevance of rafts for glutamate receptor function, different subunits of NMDARc (GluN1A, GluN2A, GluN2B) and AMPARc (GluA1, 2/3 and 4) have been localized to these membrane domains (Hering et al., 2003; Allen et al., 2007). Because of their localization in spines and importance for postsynaptic excitatory transmission we will next comment examples of particular lipids influencing AMPARc and NMDARc function.

### Lipid influence on AMPARc

AMPARc mediate most excitatory transmission in the brain, and their regulated addition and removal from the postsynaptic membrane leads to long lasting forms of synaptic plasticity such as LTP and LTD (Malinow and Malenka, 2002). In addition, AMPARc continuously cycle in and out the synaptic membrane independently of synaptic activity. This constitutive trafficking involves both exocytic delivery from intracellular compartments (Gerges et al., 2006), fast exchange with surface extra-synaptic receptors via lateral diffusion (Tardin et al., 2003) and internalization of the displaced receptors by endocytosis, which is essential to sustain LTD (Carroll et al., 1999; Beattie et al., 2000). Although for most of these effects the specific molecular mechanism has not yet been elucidated, experimental modulation of lipids in brain slices or cultured neurons lead to changes in AMPARc localization and electrophysiological behavior. As we next describe, some of these effects received *in vivo* confirmation in mouse models with genetically or experimentally altered lipid content.

Cholesterol depletion reduces early-AMPA-mediated calcium influx (Frank et al., 2008). It was proposed that this effect was not due to a direct influence on the AMPARc channel kinetics but to altered surface expression of at least a subpopulation of AMPARc. Indeed, cholesterol levels have been shown to modulate AMPARc surface mobility (Renner et al., 2009) as well as endosomal dynamics (Hering et al., 2003; Hou et al., 2008). The loss of cholesterol in synaptic membranes of aged neurons impairs AMPARc internalization due to PI3K/Akt activation, which precludes Akt dephosphorylation required for GSK3β activation-mediated glutamate receptor endocytosis (Martin et al., 2014). Quantum-dot-based single molecule tracking analysis showed that reduction of cholesterol also affects GluR2-AMPARc lateral diffusion. These alterations lead to the impaired LTD typical of old neurons. Consistently, increasing cholesterol levels *in vitro* or *in vivo* by chronic infusion of the lipid restores LTD and cognitive deficits in the old mice (Martin et al., 2014).

Little is known about the influence of membrane shingolipids in AMPARc function. A positive role for the signaling sphingolipid Sphingosine-1-phosphate (S1P) in AMPARc-mediated miniature excitatory postsynaptic currents has been reported in hippocampal slices. Inhibition of sphingosine kinase (SphK) impaired LTP that was fully restored by S1P addition. Consistently, mice lacking SphK show poor memory performance (Kanno et al., 2010). However, it has been proposed that these effects do not take place in spines but correlate with the S1P-induced translocation of S1P receptors to presynaptic terminals thereby facilitating S1P receptor-mediated signals towards glutamate release.

Different studies indicate that AMPARc trafficking depends on PIP metabolism (Figure 3). Direct association of AMPARc with PI3K is required for receptor cell surface insertion and expression during LTP (Man et al., 2003). It has been proposed that the mobility of synaptic but not extra-synaptic AMPA receptors during LTD requires PIP3 depletion (Arendt et al., 2010). Hence, down-regulation of PIP3, either by overexpression of its pleckstrin homology (PH) domain or by inhibiting PI3K, impairs PSD-95 accumulation in spines and promotes AMPARc mobility leading to their migration from the postsynaptic density, where excitatory transmission occurs, towards the perisynaptic membrane within the spine, enabling synaptic depression (Arendt et al., 2010). PIP3 effects are specific for synaptic AMPARc, since it does not affect NMDARc nor extrasynaptic AMPARc. Given PIP3 contribution to the accumulation of PSD95 at spines, it has been proposed that the lipid favors AMPARc retention via modulation of the PSD95 synaptic scaffold. Complementary, enhancement of PTEN lipid phosphatase activity, which turns PIP3 into PIP2, is able to drive depression of AMPA receptor-mediated synaptic responses. This activity is specifically required for NMDARc-dependent LTD but not for LTP or mGluR-dependent LTD (Jurado et al., 2010). Further turnover of PIP2 by PLC favors synaptic actin depolymerization and PSD95 degradation, also contributing to the reduction of surface AMPARc expression and spine remodeling (Horne and Dell’Acqua, 2007).

### Lipid influence on NMDARc

NMDARc are heterotetrameric ion channels directly implicated in LTP and LTD being the predominant molecular device for controlling synaptic plasticity and memory function (Bashir et al., 1991; Cui et al., 2004; Li and Tsien, 2009). Studies performed with purified NMDARc reconstituted in liposomes showed that membrane stretch reduce Mg^2+^ blockade of NMDA channel enhancing ion currents (Kloda et al., 2007). As these results were obtained in a minimal system that lacks cellular proteins, they unambiguously demonstrate that mechanical deformation of the lipid bilayer is sufficient to modulate the gating properties of NMDA channels (Piomelli et al., 2007). Experimental evidence suggests that as much as 60% of NMDARc are located in lipid rafts (Besshoh et al., 2005), which by virtue of their particular physical properties might dynamically regulate NMDARc subunit composition and trafficking. In support of this possibility, changes in cholesterol content inhibit NMDA-stimulated influx of calcium in hippocampal cells in culture (Frank et al., 2004). Cholesterol reduction redistributes the NMDARc GluN2B subunit, from rafts to non-raft membrane fractions (Abulrob et al., 2005). On the other hand, the cholesterol metabolite 24(S)-hydroxycholesterol has been recently identified as a potent and highly selective positive modulator of NMDARc. This hydroxysterol enhances NMDARc currents and LTP and restores behavioral and cognitive deficits in rodents treated with NMDARc channel blockers (Paul et al., 2013). It appears that the mechanism underlying these effects does not involve receptor insertion or transcription but direct binding of the lipid to a modulatory site in the receptor.

Dysregulation of brain sphingolipid balance following inhibition of NSM alters the subunit composition of NMDARc that might account for memory impairment following long-term inhibition of NSM (Tabatadze et al., 2010). NSM activity modulates the phosphorylation of the NMDARc subunit GluN1 on serine 896 promoting the clustering of these modified subunits into lipid rafts. It has been proposed that the rapid and focal generation of ceramide upon NSM activation shifts the composition of membrane lipids to bring into close proximity GluN1 and its kinases PKC and PKA (Wheeler et al., 2009). However, it is not clear if these events are the result of lateral diffusion of the kinases and membrane docked receptors or a translocation to the plasma membrane.

More work is needed to determine the influence of PIPs in NMDARc. The already mentioned observation that activation of these receptors recruits the PIP3 degrading enzyme, PTEN, to dendritic spines mediating NMDA dependent but not mGluR dependent LTD (Jurado et al., 2010), indicates a close relationship between PIP dynamics and NMDARc function. Moreover, blockade of the PIP3 synthesizing enzyme PI3K impairs forms of memory formation and LTP in the hippocampus (Chen et al., 2005). Whether and how this affects NMDARc is not known.

## Concluding remarks

We are still far from having a detailed picture of how lipids participate in dendritic spine physiology. However, research in recent years has started to unveil that they are not simple structural bystanders but play relevant roles in neurotransmission, through the control of spine architecture and by modulating neurotransmitter receptor function. As key components of postsynaptic membranes, lipids affect synaptic plasticity by shaping the membrane and modulating the levels, compartmentalization, interactions, trafficking and signaling properties of many proteins that are essential for synaptic function. By these means lipids regulate glutamate receptor function and actin cytoskeleton dynamics, which are instrumental features for postsynaptic plasticity. The application to the study of synapses of new generation fluorescent probes to label lipids, modified toxins that specifically identify them, different kinds of super-resolution microscopy and more sensitive quantitative methodologies will allow us to further dissect how spine lipids precisely function. As we know more on spine lipids the traditional view of the static role for these molecules fades away and is replaced by that of a remarkable dynamism. The activity of lipid metabolic enzymes at dendritic spines guarantees this dynamism. Some of these enzymes are constitutive components of these structures that change activity or get closer to their substrates upon stimulation. Others find different ways to reach the spine membrane when required. Deep insight on the role of lipids in dendritic spines and on how lipid pathways are topologically and temporally regulated will help to understand how we learn and keep our memories. Moreover, this will unveil the reasons behind the cognitive impairment occurring during aging and in diseases like many genetic lipidosis and neurodegenerative disorders where brain lipid imbalances have been reported. These investigations could yield novel therapeutics relying on lipid based drugs that readily cross the blood-brain barrier.

## Conflict of interest statement

The authors declare that the research was conducted in the absence of any commercial or financial relationships that could be construed as a potential conflict of interest.
